# Selection of laboratory assays for reliable assessment of complement-dependent cytotoxicity: impact of assay choice on CDC quantification

**DOI:** 10.3389/fimmu.2026.1786368

**Published:** 2026-03-12

**Authors:** Alan Majeranowski, Grzegorz Stasiłojć, Nadia Panasiuk, Marcin Okrój

**Affiliations:** Intercollegiate Faculty of Biotechnology, University of Gdańsk and Medical University of Gdańsk, Gdańsk, Poland

**Keywords:** complement system, flow cytometry, immunotherapy, propidium iodide, rituximab

## Abstract

Complement-dependent cytotoxicity (CDC) results from cell lysis induced by the membrane attack complex (MAC), a pore-forming structure assembled at the terminal stage of the complement cascade that disrupts membrane integrity and causes osmotic cell death. Several methodological approaches are available to assess CDC efficacy *in vitro*, including (i) dye influx assays that report complement-mediated membrane permeabilization (e.g., propidium iodide staining), (ii) dye-release assays using preloaded fluorescent probes (e.g., calcein-AM), and (iii) assays based on cellular metabolic activity (e.g., MTT, XTT, or Alamar Blue) or ATP content (e.g., CellTiter-Glo). Because complement acts rapidly, often killing target cells within seconds to minutes, and because some staining procedures are cytotoxic or require time-consuming steps that may introduce artefacts, selecting an appropriate, high-throughput assay is not trivial. In this study, we evaluated representative methods from each assay class (dye influx, dye release, and metabolic readouts) to measure CDC triggered by the therapeutic anti-CD20 antibody rituximab in two human lymphoma cell lines expressing the CD20 antigen. Based on our findings, we discuss the strengths and limitations of each approach, with particular emphasis on their susceptibility to false-positive and false-negative results.

## Introduction

The complement system was first described over 100 years ago (1), but interest in it has markedly increased over the past two decades. This renewed attention stems from the recognition that complement is not only a defender against pathogens but also a regulator of tissue homeostasis and metabolism, a key effector mechanism for numerous immunotherapeutics, and an important contributor to tissue regeneration (2). Regardless of the initiating trigger or pathway, activation of the complement system ultimately results in assembly of the membrane attack complex (MAC) on the target cell’s membrane. MAC is composed of a C5b fragment associated with C6, C7, C8, and multiple C9 molecules (3), which together form a pore that induces osmotic cell lysis. This process, known as complement-dependent cytotoxicity (CDC), is measurable, and CDC readouts are commonly employed to evaluate the *in vitro* efficacy of complement-activating drugs (4, 5) to assess the complement-activating potential of anti-HLA antibodies in transplant recipients (6, 7), and to determine complement activity in patient sera (8, 9). CDC assays can be performed using a variety of methods that employ different detection strategies, several of which overlap with those used in general cytotoxicity assays. However, an important consideration is the rapid kinetics of complement activity. As demonstrated by Cook et al. in a B-cell lymphoma model, CDC can be completed within 60–90 s at high serum concentrations (25%–50%), depending on the complement-activating capacity of the antibody (10). These observations are consistent with our previous findings showing that (i) in a model of antibody-sensitized sheep erythrocytes incubated in low serum concentrations (0.25%–1%), formation of complement convertases, key enzymatic complexes that amplify the cascade and directly lead to MAC assembly, becomes detectable as early as 15–30 s, peaks within the next 1–5 min, and declines after 20–30 min, with higher serum concentrations producing earlier activity peaks (11), and (ii) in a model using human Ramos lymphoma cells (substantially more resistant to complement than erythrocytes (12)) sensitized with anti-CD20 antibody and incubated in 10% serum, convertase activity begins after 30 s, peaks within the next few minutes, and returns to baseline by approximately 20 min (13, 14). Artefact-free measurement of CDC requires a properly defined time window, as prolonged exposure to active complement and sublytic concentrations of MAC may induce secondary cellular responses. These include Ca^2+^ influx, mitochondrial damage, inflammasome activation, cytochrome c release, and caspase activation, ultimately leading to apoptotic cell death (15). Moreover, some therapeutic antibodies commonly used in CDC assays can independently trigger apoptosis, which unfolds over several dozen minutes (16). Therefore, to reliably quantify CDC alone, either the experimental timing must be adjusted to the actual serum conditions or a detection method with a rapid and straightforward readout should be selected. This consideration becomes especially important when processing multiple samples, as excessive delay between measurements may distort the results. Consequently, methods that enable simultaneous readout of all samples are preferable.

The most common assay based on the dye-influx strategy is the propidium iodide (PI) test, often combined with annexin V staining to distinguish early apoptotic cells. Cells permeabilized by MAC take up PI, which binds to DNA and emits fluorescence upon excitation. When analyzed by flow cytometry, the operator can gate on objects of defined size and granularity, allowing discrimination between intact cells and debris. However, this feature also represents a limitation: PI-based cytometric readouts exclude cells that have already disintegrated due to complement-mediated cytotoxicity. This, together with the inherently non-simultaneous nature of flow-cytometric measurements, constitutes a major drawback of the method. To estimate the extent of false-negative results, we therefore performed additional co-validation by cytometrically counting the total number of cells remaining after completion of the CDC assay. Metabolic assays have long been dominated by the MTT test. However, this assay is suboptimal because, on one hand, mitochondrial enzymatic activity can be influenced by human serum albumin (17), and on the other hand, the readout requires a time-consuming solubilization step to dissolve formazan crystals, which themselves are cytotoxic (18). For these reasons, we selected PrestoBlue HS^®^ as our metabolic assay. This test relies on the reduction of resazurin by mitochondrial enzymes, yields non-toxic products, and provides a relatively rapid readout within approximately 5-10 min (19). Additionally, we employed a CellTiter-Glo assay based on bioluminescent detection of intracellular ATP, a reliable marker of cell viability (20). As a representative dye-release assay, we selected the Calcein AM test. In this approach, viable cells actively uptake Calcein AM from the surrounding medium and convert it intracellularly into a fluorescent derivative, which is released upon MAC-induced membrane permeabilization and can be quantified in the supernatant (21). Each assay was evaluated using Raji and Ramos cells, two CD20-positive human lymphoma cell lines that are sensitive to anti-CD20 therapeutic antibodies (12).

## Methods

### Cell lines

Human CD20+ human lympoma cell lines Ramos and Raji were purchased from ATCC and cultured in RPMI 1640 with L-glutamine (ATCC) and 10% FBS (ATCC) at 37°C/5% CO_2_ and used within 10 passages at >95% viability.

### Human serum

Normal human serum (NHS) was prepared from blood collected from healthy volunteers upon written informed consent. This study was performed in line with the principles of the Declaration of Helsinki. Approval was granted by the Local Bioethical Committee at the Medical University of Gdańsk (approval number NKBBN/138/2018). Blood collection, sample handling, and storage were performed as described previously (22). Heat-inactivated normal human serum (Δ NHS) was prepared from NHS by incubation at 56°C for 30 min followed by centrifugation at 5.000×G for 3 min and collection of supernatant.

### CDC assays

Calcein release assay was performed in a way that cells were diluted to 1 million per ml in PBS buffer with Ca^++^/Mg^++^ (Corning) and stained with 1 μg/ml of Calcein AM (Sigma) for 15 min at standard culture conditions. Leftovers of calcein were washed out with PBS buffer, and 2 × 10^5^ cells were pelleted onto V-shaped microplate wells and overlaid with 50 μl of indicated sample content (combinations of 10% NHS/Δ NHS, 50 μg/ml rituximab, or PBS buffer). After an incubation period of 30 or 90 min, another 50 μl of PBS buffer was added and cells were centrifuged at 500×G for 3 min. 80 μl of supernatant was transferred to a black 96-well plate, and fluorescence at λ_ex_=490/λ_em_=520 nm was recorded by a Synergy H1 reader (BioTek). Signal from the cells lysed with 30% DMSO was considered 100% (full) lysis. Metabolic assay with PrestoBlue reagent was performed in the same way, but cells were not stained with Calcein AM. Instead, 7.5 µl of reagent was added 5 min before the end of incubation period and the fluorescence at λ_ex_=490/λ_em_=520 nm was recorded. For the CellTiter-Glo assay, supernatants were removed at the end of the incubation period, and cell pellets were resuspended in 50 µl PBS. Subsequently, 50 µl of freshly prepared CellTiter-Glo^®^ reagent (Promega, cat. # G7570) was added to each well. After shaking for 2 min at 600 rpm, lysed cell suspensions were transferred to a white luminescence assay plate and incubated at room temperature in the dark for 10 min. Luminescence was then measured in each well. For cytometric analysis of PI intake, cells not stained with calcein-AM were used. After the incubation period, cells for the positive control, instead of DMSO treatment, were treated for 5 min with 2.5 μl of T buffer from ADAM AccuStain Solution kit (NanoEnTek), which contains a non-ionic detergent and PI. In all other sample conditions, 2.5 µl of N buffer from the same kit, containing PI but no detergent, was added after the incubation period, and cells were stained for at least 5 min before cytometric acquisition (CytoFlex3, Beckman Coulter). The gating strategy, including exclusion of debris, non-cellular events, and cell doublets or multiplets as well as classification of PI-low and PI-high populations, is presented in **Supplementary Figure 1**. Additionally, the total event number within the gate acquired in 80 s (volume of 40µl) was recorded.

## Results and discussion

**Figure 1:**
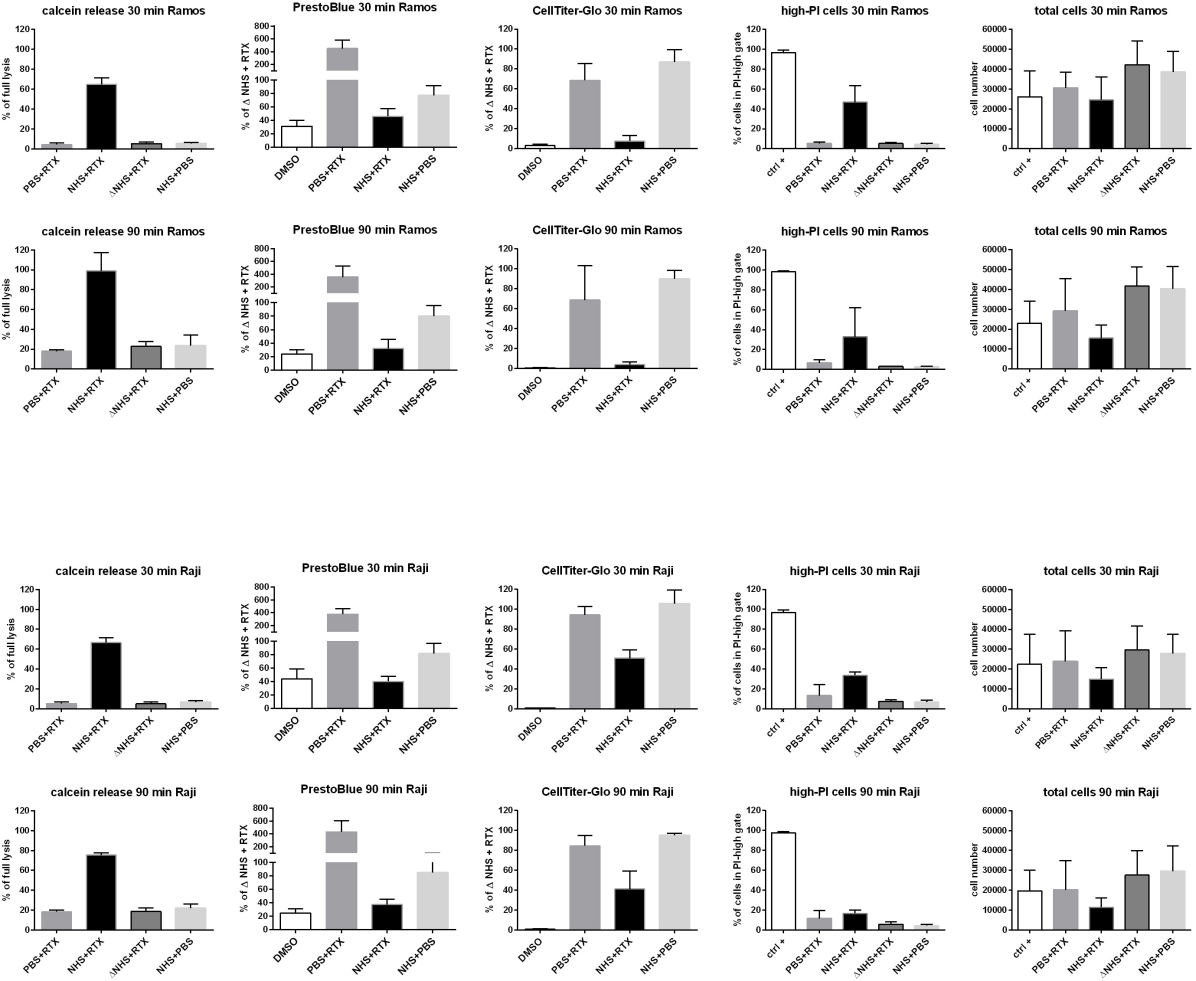
Readouts of CDC assays performed on Raji and Ramos cells challenged with rituximab. Ramos (upper panels) and Raji (lower panels) cells were challenged with 50 µg/ml rituximab in 10% normal human serum (NHS) for 30 min (upper rows) or 90 min (lower rows). Readouts of the Calcein release assay (first column from the left), PrestoBlue HS assay (second column), CellTiter-Glo assay (third column), PI uptake (fourth column), and total cell counts obtained from the PI-uptake experiments (fifth column) are shown. Data are presented as means of three independent experiments, each performed in technical duplicates. Error bars indicate standard deviation. RTX, rituximab; PBS, phosphate buffer with Ca^++^/Mg^++^; ΔNHS, heat-inactivated normal human serum; DMSO, dimethyl sulfoxide; ctrl+ - positive control in cytometric assays (see methods).

The expected experimental outcomes were as follows: (i) a robust CDC readout from CD20^+^ cells exposed to rituximab in the presence of NHS and (ii) minimal CDC from cells treated with NHS alone or with rituximab combined with heat-inactivated NHS or PBS. However, (iii) a minor contribution of rituximab-induced direct cell death cannot be excluded, particularly at longer incubation times, as previously reported (23, 24). To assess CDC, we employed four complementary assays representing distinct methodological approaches in two cell lines known to be sensitive to the anti-CD20 therapeutic antibody rituximab, a potent activator of the classical complement pathway (12, 25). Calcein-loaded Ramos and Raji cells released approximately 70% of the dye after a 30-min challenge with rituximab and NHS (**Figure 1**), whereas background release did not exceed 10%. Extending the incubation to 90 min resulted in a markedly increased background signal, reaching ~30% of maximal lysis, accompanied by a concomitant rise in CDC to nearly 100% in Ramos cells and 75% in Raji cells.

The use of the PrestoBlue HS and the CellTiter-Glo reagents required a modification of the quantification strategy. In contrast to the Calcein release assay, this method assumes that the detected signal originates from metabolically active, viable cells. Consequently, a different condition was selected as the positive control: cells treated with rituximab in the presence of complement-inactive serum (ΔNHS + RTX). CDC was then calculated as the difference between this reference condition and cells incubated with rituximab and active serum. Under these conditions, PrestoBlue assay revealed that Ramos cells retained approximately 45% of the reference signal after 30 min and 30% after 90 min, corresponding to 55% and 70% CDC, respectively. Under analogous conditions, Raji cells retained approximately 40% of the reference signal after 30 min (60% CDC), which decreased slightly to ~35% (65% CDC) after 90 min. However, a substantial assay inertia of the PrestoBlue readout must be taken into account, as illustrated by the response of DMSO-treated cells. Although DMSO caused extensive physical disruption of the cells, evidenced by the absence of a detectable pellet after centrifugation in a V-shaped well plate, approximately 30%–45% of the reference signal was still detectable after 30 min of incubation, decreasing to ~25% at 90 min. These observations indicate a delayed loss of metabolic signal following cell destruction and suggest the presence of a false-negative component in the NHS + RTX condition, an effect that can be particularly pronounced at shorter incubation times. Consequently, the extent of CDC in this setting is likely underestimated when inferred solely from the PrestoBlue assay. Another limitation of the PrestoBlue assay is its susceptibility to interference by human serum, which was clearly evident from the PBS + RTX condition, exhibiting a several-fold higher signal than other negative controls. Given the known mechanism of CDC and the established biological activity of rituximab, there is no mechanistic rationale for such an effect. Instead, this observation is most likely attributable to partial inhibition or quenching of the PrestoBlue signal by serum components (a phenomenon well-known for other metabolic assays (17)), resulting in an apparent difference relative to the remaining negative control conditions.

Out of the two metabolic assays, much better performance was obtained for the CellTiter-Glo technique. First, very low background readout below 3% was observed irrespectively of the incubation period. Then, unlike in PrestoBlue, no serum interference with signal was observed. The assay revealed 8% and 3% of ATP content in Ramos cells after 30- and 90-min incubation with rituximab and NHS vs. the control condition with ΔNHS (that corresponds to 92% and 97% CDC), respectively. Analogous readouts for Raji cells were 50% of ATP content after 30 min (50% CDC) and 41% of ATP content after 90 min (59% CDC).

Analysis of the dye uptake assay, based on the percentage of PI-positive cells, indicated that CDC in Ramos and Raji cells reached approximately 45% and 35%, respectively, after 30 min of incubation. However, somewhat unexpectedly, prolongation of incubation to 90 min resulted in a decrease in the fraction of PI-positive cells in both cell lines: from 45% to 30% in Ramos cells and from 30% to 15% in Raji cells. It appears highly unlikely that, in the presence of high concentrations of rituximab and serum, cells recovered from extensive MAC formation, particularly since no other assay supported such a scenario. Rather, this paradoxical reduction suggests that extensive physical disintegration of the cells rendered them undetectable by flow cytometry. Such a scenario became plausible when additional insight was obtained by quantifying the total number of events recorded by the flow cytometer across all gates, including both PI-low and PI-high populations. Consistent with our interpretation, detergent-treated cells (ctrl + sample) were almost exclusively detected within the PI-high gate, in contrast to cells treated with rituximab and serum. However, even in these detergent−treated positive control samples, the total number of detectable events decreased compared with the negative control conditions (ΔNHS + RTX and NHS + PBS, which both yielded comparable event counts), indicating that a substantial fraction of PI−permeable cells is lost before the selected time points. Similarly, a consistent loss of detectable cells was observed in the NHS + RTX samples. For instance, after 90 min, only ~40% of Raji cells were recorded in the NHS + RTX condition compared with ΔNHS + RTX. Given that approximately 60% of cells were no longer detected and that ~15% of the remaining cells were PI-positive, the overall extent of CDC can be estimated at ~66%. Notably, this corrected value lies between the estimates obtained using the calcein-release and PrestoBlue assays, whereas the raw CDC value of 15% inferred from PI uptake alone is inconsistent with both the earlier time point (which would require recovery from PI permeability) and the results obtained using complementary assay approaches.

Interestingly, a substantial proportion of cells was lost not only in detergent-treated samples (ctrl + condition) or rituximab + serum samples (NHS + RTX) but also in cells treated with rituximab in PBS (RTX + PBS condition) when experiments aimed at determining total cell numbers were performed. We attribute this effect to differences in cell recovery from the wells prior to flow cytometric analysis under serum-free vs. serum-supplemented conditions. No substantial differences in the doublet/multiplet exclusion ratios were observed between conditions (data not shown), indicating that the observed cell loss was unlikely due to aggregation. Instead, the effect most likely reflects a technical issue related to cell pellet adherence or compactness, which reduced the efficiency of cell transfer to flow cytometry tubes.

## Conclusions

Collectively, our findings demonstrate that, despite its widespread use and methodological advantages, such as the possibility of simultaneous analysis of cell surface markers and discrimination between target and non-target populations (26–29), flow cytometric assessment of propidium iodide (PI) uptake without additional cell quantification does not provide a reliable or quantitative measure of complement-dependent cytotoxicity (CDC). Under certain experimental conditions, reliance solely on PI uptake may lead to substantial underestimation of complement-mediated lysis. This limitation was clearly illustrated by the paradoxical reduction in the percentage of PI-positive cells observed after 90 min compared with 30 min of incubation, a phenomenon that was further corroborated by a decrease in the total number of detectable cells across all cytometric gates. To the best of our knowledge, this specific limitation of PI-based CDC assessment has not been explicitly highlighted in the existing literature. Moreover, our findings underscore that flow cytometry–based CDC measurements are vulnerable to additional technical confounders, such as inefficient or condition-dependent cell recovery, which may substantially distort quantitative readouts if not carefully controlled.

In addition, our results indicate that some metabolic viability assays, exemplified here by PrestoBlue, are susceptible to signal quenching by serum. This represents a critical drawback in CDC assays, where serum simultaneously serves as both the source of complement activity and a potential confounder of assay readouts. Because PrestoBlue and other widely used metabolic assays, such as MTT or XTT, report cellular metabolic activity rather than membrane integrity, their signals may persist long after irreversible cell damage has occurred. This was evident from the residual signal detected in DMSO-lysed cells, which did not fully decline even after a 90-min incubation period. In contrast, the ATP-based CellTiter-Glo assay was not affected by such artifacts. Indeed, DMSO-lysed cells displayed less than 1% of the control signal after 90 min, as expected when complete physical disintegration occurs prior to measurement. Moreover, no serum-dependent signal quenching was observed. A slight decrease in the PBS + RTX control, particularly in Ramos cells, may instead reflect pellet adherence or compactness issues under serum-free conditions, similar to those discussed for cytometric cell counting. Notably, the use of ATP measurement as a surrogate for CDC may theoretically lead to misinterpretation, since sublytic MAC deposition can perturb mitochondrial function and influence intracellular ATP levels before overt membrane rupture occurs (15, 30). Thus, ATP-based assays may capture early complement-induced injury rather than strictly reflecting terminal cell lysis. Interestingly, CellTiter-Glo revealed rituximab-induced CDC in Ramos cells at levels substantially higher than those detected by other assays (with the exception of PI incorporation alone), whereas CDC in Raji cells remained within a comparable range but was still the lowest among all readouts. We speculate that this discrepancy may reflect differences in the susceptibility of Ramos and Raji cells to sublytic MAC-mediated stress. Nevertheless, ATP-based measurements appear more robust than PrestoBlue in our experimental setting, as the overall results were consistent with theoretical expectations. Taken together, these findings indicate that, despite the potential influence of sublytic MAC on cellular ATP homeostasis, CellTiter-Glo provides a sensitive and quantitative readout of CDC under the conditions tested.

Among the assays evaluated in this study, Calcein release likewise emerged as a robust and quantitative method for bulk CDC assessment, offering results that were largely in agreement with ATP-based measurements. However, increased nonspecific dye leakage at prolonged incubation times should be considered an inherent limitation of this approach. Thus, both CellTiter-Glo and Calcein release assays appear well suited for CDC quantification, provided that their respective constraints are taken into account. Regardless of the assay employed, accurate interpretation of CDC measurements critically depends on careful consideration of assay timing, as well as the inclusion of appropriate controls, including no-antibody, no-complement, and full-lysis conditions.

## Data Availability

The original contributions presented in the study are included in the article/**Supplementary Material**. Further inquiries can be directed to the corresponding author.

## References

[1] Cavaillon J-M Sansonetti P Goldman M . 100th anniversary of Jules Bordet’s Nobel Prize: Tribute to a founding father of immunology. Front Immunol. (2019) 10:2114. doi: 10.3389/fimmu.2019.02114. PMID: 31572361 PMC6749103

[2] West EE Woodruff T Fremeaux-Bacchi V Kemper C . Complement in human disease: Approved and up-and-coming therapeutics. Lancet. (2024) 403:392–405. doi: 10.1016/S0140-6736(23)01524-6. PMID: 37979593 PMC10872502

[3] Merle NS Church SE Fremeaux-Bacchi V Roumenina LT . Complement system part I - Molecular mechanisms of activation and regulation. Front Immunol. (2015) 6:262. doi: 10.3389/fimmu.2015.00262. PMID: 26082779 PMC4451739

[4] Stasiłojć G Österborg A Blom AM Okrój M . New perspectives on complement mediated immunotherapy. Cancer Treat Rev. (2016) 45:68–75. doi: 10.1016/j.ctrv.2016.02.009. PMID: 26994325

[5] Ajona D Cragg MS Pio R . The complement system in clinical oncology: Applications, limitations and challenges. Semin Immunol. (2024) 77:101921. doi: 10.1016/j.smim.2024.101921. PMID: 39700788

[6] Wong ZE Downing J De Santis D Halse M Bruce S Truong L . C3d-binding assay for the detection of complement activating HLA antibodies: A useful tool for allocation to highly sensitised recipients in the post-CDC era? HLA. (2023) 102:13–27. doi: 10.1111/tan.15010. PMID: 36851856

[7] Putheti P Liwski RS Jindra PT . Reducing number of laboratories performing complement dependent cytotoxicity crossmatching: Reasons and conclusions. Hum Immunol. (2022) 83:467–75. doi: 10.1016/j.humimm.2022.02.001. PMID: 35183390

[8] Felberg A Taszner M Urban A Majeranowski A Jaskuła K Jurkiewicz A . Monitoring of the complement system status in patients with B-cell Malignancies treated with rituximab. Front Immunol. (2020) 11:584509. doi: 10.3389/fimmu.2020.584509. PMID: 33329558 PMC7710700

[9] Middleton O Cosimo E Dobbin E McCaig AM Clarke C Brant AM . Complement deficiencies limit CD20 monoclonal antibody treatment efficacy in CLL. Leukemia. (2015) 29:107–14. doi: 10.1038/leu.2014.146. PMID: 24787488

[10] Cook EM Lindorfer MA van der Horst H Oostindie S Beurskens FJ Schuurman J . Antibodies that efficiently form hexamers upon antigen binding can induce complement-dependent cytotoxicity under complement-limiting conditions. J Immunol. (2016) 197:1762–75. doi: 10.4049/jimmunol.1600648. PMID: 27474078 PMC4991250

[11] Blom AM Volokhina EB Fransson V Strömberg P Berghard L Viktorelius M . A novel method for direct measurement of complement convertases activity in human serum. Clin Exp Immunol. (2014) 178:142–53. doi: 10.1111/cei.12388. PMID: 24853370 PMC4360204

[12] Okroj M Eriksson I Österborg A Blom AM . Killing of CLL and NHL cells by rituximab and ofatumumab under limited availability of complement. Med Oncol. (2013) 30:759. doi: 10.1007/s12032-013-0759-5. PMID: 24198205

[13] Kuźniewska A Majeranowski A Henry S Kowalska D Stasiłojć G Urban A . The acquisition of complement-dependent cytotoxicity by the type II anti-CD20 therapeutic antibody obinutuzumab. Cancers (Basel). (2023) 16:49. doi: 10.3390/cancers16010049. PMID: 38201478 PMC10778491

[14] Stasiłojć M Stasiłojć G Kuźniewska A Rodriguez de Córdoba S Okrój M . A cell-based assay to measure the activity of the complement convertases. Kidney Int Rep. (2024) 9:2260–8. doi: 10.1016/j.ekir.2024.04.058. PMID: 39081762 PMC11284395

[15] Triantafilou K Hughes TR Triantafilou M Morgan BP . The complement membrane attack complex triggers intracellular Ca2+ fluxes leading to NLRP3 inflammasome activation. J Cell Sci. (2013) 126:2903–13. doi: 10.1242/jcs.124388. PMID: 23613465

[16] Edelmann J Dokal A Holzmann K Britton DJ Vilventhraraja E Smith RJ . Rituximab and obinutuzumab induce direct B-cell death via B-cell receptor (BCR) signaling, but rituximab elicits stronger BCR-derived pro-survival signals diminishing apoptosis. Blood. (2021) 134:1579. doi: 10.1182/blood-2019-128704. PMID: 41496790

[17] Funk D Schrenk H-H Frei E . Serum albumin leads to false-positive results in the XTT and the MTT assay. Biotechniques. (2007) 43:178–82. doi: 10.2144/000112528. PMID: 17824385

[18] Lü L Zhang L Wai MSM Yew DTW Xu J . Exocytosis of MTT formazan could exacerbate cell injury. Toxicol Vitro. (2012) 26:636–44. doi: 10.1016/j.tiv.2012.02.006. PMID: 22401948

[19] Luzak B Siarkiewicz P Boncler M . An evaluation of a new high-sensitivity PrestoBlue assay for measuring cell viability and drug cytotoxicity using EA.hy926 endothelial cells. Toxicol Vitro. (2022) 83:105407. doi: 10.1016/j.tiv.2022.105407. PMID: 35659575

[20] Peternel L Kotnik M Prezelj A Urleb U . Comparison of 3 cytotoxicity screening assays and their application to the selection of novel antibacterial hits. J Biomol Screen. (2009) 14:142–50. doi: 10.1177/1087057108329452. PMID: 19196697

[21] Stasiłojć G Felberg A Urban A Kowalska D Ma S Blom AM . Calcein release assay as a method for monitoring serum complement activity during monoclonal antibody therapy in patients with B-cell Malignancies. J Immunol Methods. (2020) 476:112675. doi: 10.1016/j.jim.2019.112675. PMID: 31629742

[22] Felberg A Urban A Borowska A Stasiłojć G Taszner M Hellmann A . Mutations resulting in the formation of hyperactive complement convertases support cytocidal effect of anti-CD20 immunotherapeutics. Cancer Immunol Immunother. (2019) 68:587–98. doi: 10.1007/s00262-019-02304-0. PMID: 30725204 PMC6447516

[23] Golay J Zaffaroni L Vaccari T Lazzari M Borleri GM Bernasconi S . Biologic response of B lymphoma cells to anti-CD20 monoclonal antibody rituximab *in vitro*: CD55 and CD59 regulate complement-mediated cell lysis. Blood. (2000) 95:3900–8. doi: 10.1182/blood.V95.12.3900.012k14_3900_3908 10845926

[24] Bezombes C Grazide S Garret C Fabre C Quillet-Mary A Müller S . Rituximab antiproliferative effect in B-lymphoma cells is associated with acid-sphingomyelinase activation in raft microdomains. Blood. (2004) 104:1166–73. doi: 10.1182/blood-2004-01-0277. PMID: 15126316

[25] Winiarska M Glodkowska-Mrowka E Bil J Golab J . Molecular mechanisms of the antitumor effects of anti-CD20 antibodies. Front Biosci (Landmark Ed). (2011) 16:277–306. doi: 10.2741/3688. PMID: 21196171

[26] Jacobs DB Pipho C . Use of propidium iodide staining and flow cytometry to measure anti-mediated cytotoxicity: Resolution of complement-sensitive and resistant target cells. J Immunol Methods. (1983) 62:101–8. doi: 10.1016/0022-1759(83)90115-1, PMID: 6192174

[27] Dantas E Erra Díaz F Pereyra Gerber P Merlotti A Varese A Ostrowski M . Low pH impairs complement-dependent cytotoxicity against IgG-coated target cells. Oncotarget. (2016) 7:74203–16. doi: 10.18632/oncotarget.12412. PMID: 27716623 PMC5342046

[28] Bellosillo B Villamor N López-Guillermo A Marcé S Esteve J Campo E . Complement-mediated cell death induced by rituximab in B-cell lymphoproliferative disorders is mediated *in vitro* by a caspase-independent mechanism involving the generation of reactive oxygen species. Blood. (2001) 98:2771–7. doi: 10.1182/blood.V98.9.2771. PMID: 11675350

[29] Miller ML Finn OJ . Flow cytometry-based assessment of direct-targeting anti-cancer antibody immune effector functions. Methods Enzymol. (2020) 632:431–56 doi: 10.1016/bs.mie.2019.07.026. PMID: 32000909 PMC7000137

[30] Tegla CA Cudrici C Patel S Trippe R 3rd Rus V Niculescu F . Membrane attack by complement: The assembly and biology of terminal complement complexes. Immunol Res. (2011) 51:45–60. doi: 10.1007/s12026-011-8239-5. PMID: 21850539 PMC3732183

