# Supplementary material for: Selection of laboratory assays for reliable assessment of complement-dependent cytotoxicity: impact of assay choice on CDC quantification
Source: Front Immunol. 2026 Mar 12;17:1786368. doi: 10.3389/fimmu.2026.1786368 (PMC13017253; doi:10.3389/fimmu.2026.1786368)

Fig. S1. Example gating strategy for flow cytometry data. (A) All recorded events were plotted on FSC-A versus SSC-A, and a rectangular gate (“all”) was drawn to include the main cell population while excluding debris and non-cellular events. (B) Events within the previously mentioned gate were then plotted on FSC-H versus FSC-Width, and singlets (single-cell events, as opposed to doublets or multiplets) were selected by excluding events with increased FSC-Width at a given FSC-H. (C, D) Events within the singlet gate were displayed as a PE-A (PI) histogram, and the “low-PI” and “high-PI” regions were defined using positive control cells (C) treated with T buffer (containing PI and detergent) and cells cultured in PBS with NHS (D) as reference control.


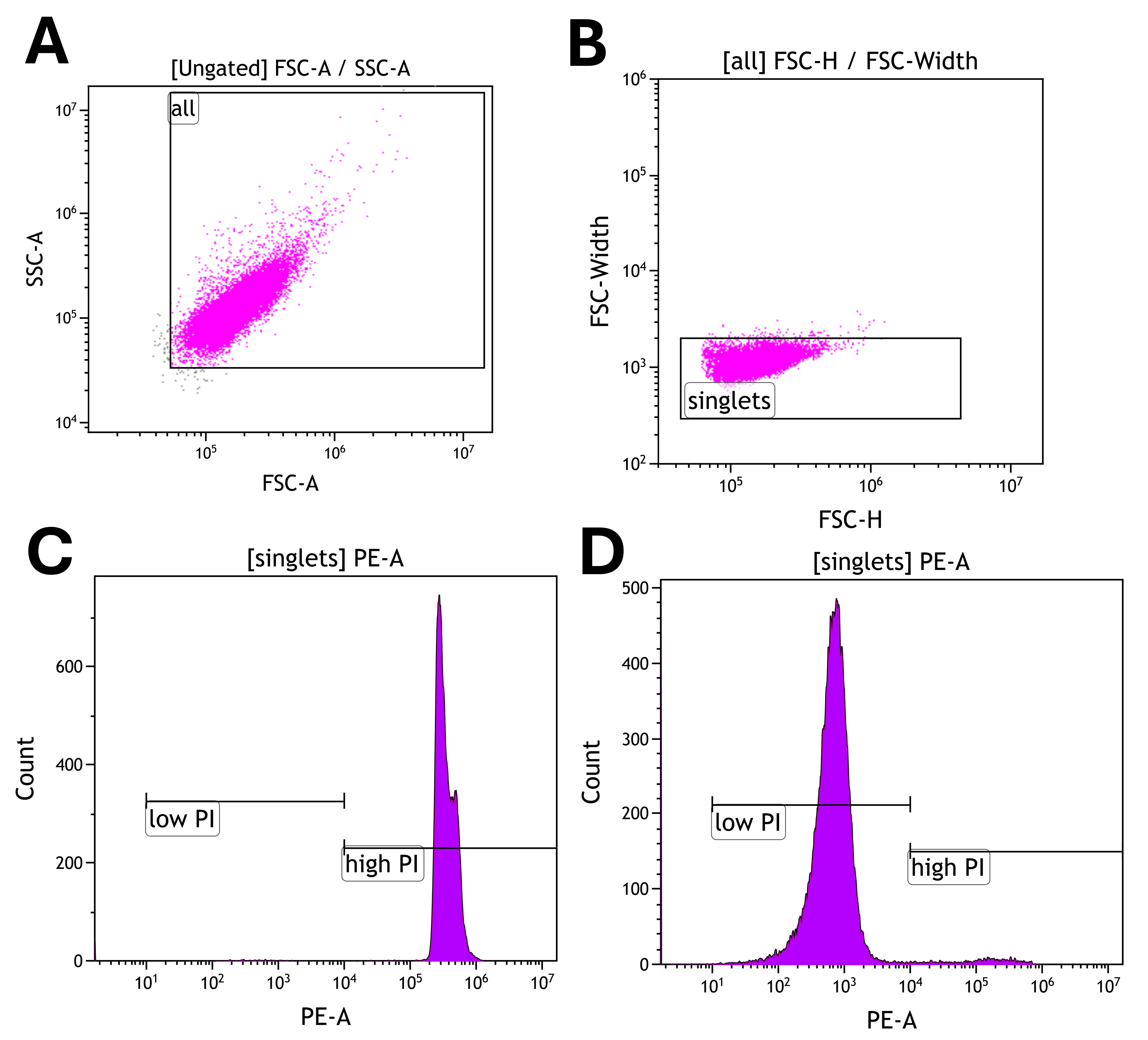

Supplement: Supplementary file 1 [file Table1.docx]
